# A Guide for Selection of Genetic Instruments in Mendelian randomisation (MR) studies of Type-2 diabetes and HbA_1c_: towards an integrated approach

**DOI:** 10.2337/db22-0110

**Published:** 2023-02-01

**Authors:** Victoria Garfield, Antoine Salzmann, Stephen Burgess, Nish Chaturvedi

**Affiliations:** 1 MRC Unit for Lifelong Health and Ageing, Institute of Cardiovascular Science, University College London; 2 Department of Public Health and Primary Care, University of Cambridge, Cambridge, UK, MRC Biostatistics Unit, University of Cambridge, UK

**Keywords:** Mendelian randomisation, diabetes, genetic variants, instrument strength, UK Biobank, HbA1c

## Abstract

This study examines the instrument selection strategies currently employed throughout the type-2 diabetes and HbA_1c_ MR literature. We then argue for a more integrated and thorough approach, providing a framework to do this in the context of HbA_1c_ and diabetes. We conducted a literature search for Mendelian randomisation studies that have instrumented diabetes and/or HbA_1c_. We also used data from the UK Biobank (N=349,326) to calculate instrument strength metrics that are key in MR studies (the F-statistic for average strength and R^2^ for total strength) with two different methods (‘Individual-level data regression’ and Cragg-Donald formula). We used a 157-SNP instrument for diabetes and a 51-SNP instrument (as well as partitioned into glycaemic and erythrocytic) for HbA_1c_. Our literature search yielded 48 studies for diabetes and 22 for HbA1c. Our UKB empirical examples showed that irrespective of, the method used to calculate metrics of strength and whether the instrument was the main one or was partitioned by function, the HbA1c genetic instrument is strong in terms of both average and total strength. For diabetes, a 157-SNP instrument was shown to have good average and total strength, but these were both substantially smaller than those of the HbA_1c_ instrument. We provide a careful set of five recommendations to researchers who wish to genetically instrument type-2 diabetes and/or HbA_1c_. MR studies of glycaemia should take a more integrated approach when selecting genetic instruments and we give specific guidance on how to do this.

## Introduction

Mendelian randomisation (MR) has markedly enhanced our ability to determine true causal nature of associations between states of diabetes (1–45) /hyperglycaemia (46–59) and presumed consequences. MR uses genetic variants as unconfounded instruments for the exposure (60). As MR has come of age in recent years alongside the advent of large-scale genome-wide association studies (GWAS), numerous genetic instruments for glycaemic traits have become available (61–65). Choosing the most appropriate instrument is one of the most important decisions when designing an MR study(66) as an ill-informed choice may lead to misleading or conflicting findings.

**Fig. 1:**
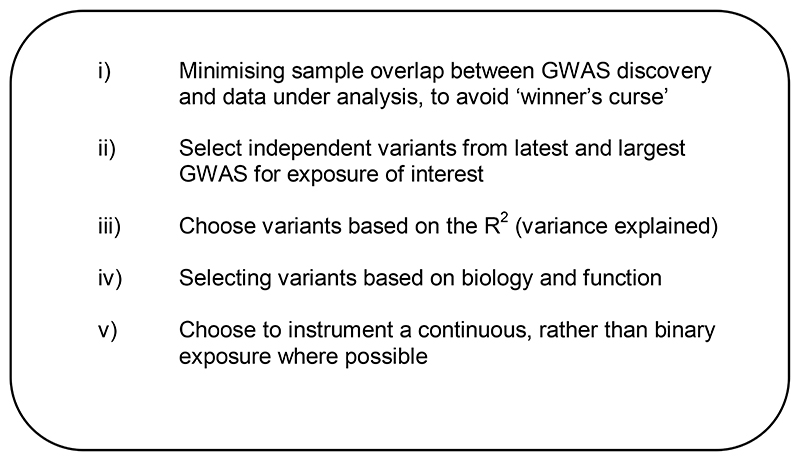
Summary of genetic instrument selection criteria in MR studies

Broadly, criteria for instrument selection (which are intrinsically linked to the core assumptions underlying MR - Fig. 1) include: i) ensuring that there is no sample overlap between the samples used in the discovery genome-wide association study (GWAS) and the data under analysis, as this helps minimise bias arising from “winner’s curse” and the use of weak instruments - (67); ii) selecting independent variants from the latest and largest GWAS for the exposure (at a threshold of p<5*10^-8^); iii) choosing variants based on the amount of variance explained in the exposure (R^2^); iv) selecting variants on the basis of biology and function; and v) deciding whether variants for a continuous, or a binary exposure are more appropriate. However, often prioritised in glycaemic MR studies are i), ii) and perhaps iii), but the remainder are not always taken into consideration. In relation to ii, we argue that bigger is not always better, as the greater the number of genetic variants, the more we increase our chances of including pleiotropic variants. This directly violates a core MR assumption (no horizontal pleiotropy: that variants for the exposure should not be associated with common confounders or directly with the outcome under study but should only associate with the outcome *via* the exposure being instrumented)(60). A balance is needed between including sufficient genetic variants to enable well-powered analyses, but not so many that pleiotropy is inevitable.

Currently few, if any journals, demand a clear explanation for choice of genetic instrument. While some determinants of choice, such as overlap with genetic instrument derivation GWAS, variant function and whether the trait is continuous or binary, may be gleaned from the manuscript without being explicit, key statistical characteristics, specifically R^2^ and F, which may make a major contribution to the power of an MR analysis, are not. Here the R^2^ is the amount of variance in the exposure that is accounted for by the selected genetic variants and generally when it comes to the R^2^, the larger the better, as this will directly contribute to the power of an MR analysis. The F-statistic provides information about the average strength of a genetic variant for the exposure of interest. An F of >10 indicates that substantial weak instrument bias is unlikely (1/F of the bias from the observational estimate) (68). Weak instrument bias is of concern in MR studies, as weak instruments can bias MR estimates towards the confounded observational estimate (68) and thus, results are not as robust as with a strong instrument.

Therefore, our overall objectives were to understand instrument selection approaches currently used in MR studies of diabetes and HbA_1c_, to present why we need integrated approaches (described below) for this and provide a framework for how this can be done in practical terms. Our specific aims were:

1. Conduct a literature search for MR studies that have instrumented type-2 diabetes and/or HbA_1c_ to understand which exposure is instrumented more frequently and whether they report metrics of instrument strength.
2. Argue for the use of integrated approaches for the selection of HbA_1c_ and type-2 diabetes genetic instruments, with recent examples from the MR literature.
3. Use empirical examples to compare the *total* and *average* strength of an HbA_1c_ genetic instrument (including partitioned by function) with a type-2 diabetes instrument to show that an HbA_1c_ instrument may be superior.
4. Provide an overall framework for how to best select instruments for HbA_1c_ and type-2 diabetes in an MR setting, considering 1 and 2.

## Making the Case for Integrated Approaches When Selecting Hba1C and Type-2 Diabetes Instruments for Use in Mr Studies

Here we highlight recent examples from the MR literature which have used HbA_1c_ and/or diabetes genetic variants in MR studies, in what we are naming “an integrated approach”. An integrated approach to genetic instrument selection is one that considers factors which are sometimes overlooked in MR studies of glycaemic traits. These include: the use of novel approaches, such as for example that of Burgess and colleagues(57) described here; more careful consideration of which exposure GWAS is used; where possible prioritising instrumentation of a continuous rather than a binary exposure; and finally, ensuring that both the variance explained (R^2^) and measures of instrument strength (F-statistic) are always calculated and presented.

### Example A. Published MR study of glycaemia and coronary heart disease using an integrated approach to HbA1c genetic instrument selection

A recent MR study by Burgess and colleagues (57) used HbA_1c_ genetic variants to investigate associations between genetically-instrumented glycaemic status and incident coronary heart disease. The authors used a novel approach to genetic instrument selection: they took 40 independent HbA_1c_ SNPs based on their associations with diabetes at genome-wide significance from a recent GWAS (64) and their association with HbA_1c_ in the 2017 MAGIC GWAS by Wheeler et al.(61). They then calculated a weighted allele score for each individual in their data (UK Biobank) whereby they multiplied each diabetes risk-increasing allele dosage by the SNP’s HbA_1c_ beta coefficient from the MAGIC GWAS. By doing so, the authors ensured that their allele score reflected average blood glucose levels, as opposed to only HbA1c or risk of diabetes. This also relates to our earlier point about selecting instruments based on biological function. Corresponding metrics for their instrument were F=144.5 and R^2^=0.018, indicating that although they had fewer variants, this was a strong instrument, both in terms of total (R^2^) and average strength (F-statistic) and thus, carried a low risk of weak instrument bias.

### Example B. Published MR study of glycaemia and cognitive/brain health

As mentioned earlier, an assumption that is often made when approaching genetic instrument selection in MR studies is that ‘bigger is better’. Therefore, researchers are likely to take as many SNPs (genome-wide significant and independent) as possible from the largest and latest GWAS. However, our own recently published MR study shows that this is not necessarily the case(69). We instrumented diabetes using both a 157- and 77-SNP genetic instrument, as we needed to try to mitigate issues of sample overlap between the GWAS for the exposure and the data under study (both UKB). Therefore, we took the 157 diabetes SNPs included in our instrument and looked them up in an older diabetes GWAS from 2014 (70). We found 77 of the diabetes SNPs (reduced number could be due to differences in coverage of imputation panels, for example) and observed that although this was an older GWAS in a different and smaller sample, the log(betas) for each SNP were comparable, even though most of the variants did not reach conventional genome-wide significance (p<5*10^-8^). When we calculated the average strength (F-statistic) of our 77-SNP instrument and compared this with the 157-SNP F-statistic they were 31 and 27, respectively. This indicates that an instrument with more genetic variants is not necessarily better in terms of average strength and the greater the number of variants, the greater the likelihood of including pleiotropic variants.

That a greater number of SNPs is not always better is also supported by recent MR studies that have instrumented body mass index (BMI)(71). The authors used an ‘older’ instrument containing 96 BMI SNPs performs well and therefore, it is perhaps unnecessary to always use an instrument with hundreds of SNPs. Larsson and colleagues showed that this BMI instrument explained 1.6% of the variance in BMI and had an F-statistic of 61 (71), while another recent MR study that instrumented BMI to understand its association with chronic kidney disease (CKD) used a 773-SNP instrument, which explained ~6% of the variance in BMI but only had an F-statistic of 23.6 (72). It is important to note that when selecting a genetic instrument for an MR study we need to balance these metrics against one another. This is because an instrument with more genetic variants has a larger R^2^ (total strength) and more power but is also more likely to include pleiotropic variants which could lead to violation of a core MR assumption. An instrument with a larger R^2^ usually has a lower F-statistic (average strength) which, if <10 will carry a greater risk of weak instrument bias.

## Methods

### Literature search for Mendelian randomisation studies that instrument type-2 diabetes and/or HbA_1c_

We were interested in how many studies have instrumented HbA_1c_ and type-2 diabetes to date, whether there is a preference for one over the other and whether they report metrics of instrument strength. Thus, we conducted a literature review in PubMed up until March 2021 (for details of our search terms and strategy see Supplemental Material S1) of MR studies that instrumented these exposures. We excluded anything that was not a research article, i.e., conference abstracts, letters, editorials, reviews, opinion pieces and commentaries. Studies that evidently did not instrument HbA_1c_ or type-2 diabetes were not included. Supplementary Material Tables 1 and 2 list all the studies for diabetes and HbA_1c_, respectively, that were included.

### Empirical examples in UK Biobank (UKB): Calculation of total (R^2^) and average strength (F-statistic) metrics for HbA_1c_ and type-2 diabetes instruments

The aim of these empirical examples was to show the reader that, a) calculating (R^2^ and) F-statistic metrics as part of an MR study is important to understand both the *total* and *average* strength of the instrument of choice and b) irrespective of whether individual- or summary-level data are used for an MR study, options for obtaining these metrics are available. We chose two approaches as there has not been any quantitative comparison of how they perform for glycaemic instruments when considering both the R^2^ and F-statistic. These methods are: ‘Individual-level data regression’ and Cragg-Donald F-statistic.

#### Sample

The UKB is a cohort of ~500,000 adults recruited across the UK general population, aged 40-69 years at baseline (2006-2010) for which more details are published elsewhere (73). For the empirical examples in the ‘Individual-level data regression’ and the Cragg-Donald method we used individual-level data from 349,326 UKB participants of white European ancestry, who had complete genotype (quality-controlled) and phenotype data (type-2 diabetes and HbA_1c_). Details of the genotype QC can be found in our previous MR paper (69). The UKB received ethical approval from the North West Multicentre Research Ethics Committee and obtained informed consent from participants.

#### Statistical analyses

Selection of type-2 diabetes and HbA_1c_ genetic instruments For both phenotypes, we used previously-described genetic instruments (69). Briefly, for type-2 diabetes the genetic instrument comprised 157 single nucleotide polymorphisms (SNPs) from a 2018 GWAS of European ancestry (74), while the 51-SNP HbA_1c_ instrument came from a 2017 trans-ethnic GWAS (61). We filtered SNPs on minor allele frequency (>0.01), used LD clumping in PLINK and p<5*10^-8^ (69). For HbA1c we also partitioned the instrument into 16 glycaemic SNPs and 19 erythrocytic SNPs (the remainder are unclassified, as per the 2017 GWAS) separately with the aim of testing whether the HbA_1c_ instrument is strong in terms of both *average* (measured by the F-statistic) and *total strength* (measured by the R^2^) when using all the SNPs, as well as when we partition it by biological function. Similarly to our previously published MR study of glycaemia and brain health/cognition/dementia outcomes, we suggest that it is worth doing three things when using an HbA1c genetic instrument: i) perform MR using all of the HbA_1c_ SNPs, ii) perform MR using only the glycaemic SNPs, iii) perform MR using only the erythrocytic SNPs.

#### Calculation of the F-statistic as a measure of average instrument strength and the R^2^ as a measure of total strength

**‘Individual-level data regression approach’:** this approach involves fitting a multivariable linear regression between SNPs and the exposure (treated as an outcome *y* here), where the relationship between the j-th SNP and the outcome *y* is evaluated while holding all the other SNPs constant. In the regression equation below *β_0_* represents the constant and *ε* the residual or error term. As with any multivariable regression the output includes the F-statistic and R^2^, which conventionally indicate the model fit and, in this case, we are likely to not be concerned with the interpretation of the coefficients of each SNP on the exposure. Linear regression can also be used when the exposure is binary (e.g., in this case, we used it for genetic liability to diabetes), whereby the coefficients and statistics represent associations on an absolute scale rather than a relative risk or odds ratio scale. Therefore, here we calculated R^2^ and the F-statistic for liability to diabetes using linear regression.

The formula is thus:

$$y=\sum_{j=1}^{J}x^{j}\beta^{j}+\beta_{0}+\varepsilon$$

**Cragg-Donald F-statistic formula:** this method uses the Cragg-Donald F-statistic formula provided in the paper by Burgess and colleagues (68) which requires a value for R^2^ (previously calculated R^2^ values were 0.028 and 0.030 for HbA_1c_, and 0.015 for diabetes), *k* (number of SNPs= 51,275 and 157) and *n* (349,326). For consistency and comparability, we kept the R^2^, *k* and *n* the same as in the ‘Individual-level data regression’ approach above. Above, we were able to calculate the R^2^, but it is sometimes the case that GWAS authors provide the R^2^ for the top SNPs which could then be used in this formula.

The Cragg-Donald formula, as outlined in Burgess 2011 (68) is:

$$F=\left(\frac{\text{n}-\text{k}-1}{\text{k}}\right)\left(\frac{R^{2}}{1-R^{2}}\right)$$

## Results

### Literature search results

Our searches yielded a total of 657 studies for diabetes, of which 609 did not instrument this phenotype and thus 48 remained. For HbA_1c_, we found a total of 77 articles, of which 55 did not instrument HbA_1c_ and were excluded, leaving 22 articles. From this literature search it was clear that many more studies currently choose to instrument type-2 diabetes over HbA_1c_.

### Results of F-statistic (average instrument strength) and R^2^ (total instrument strength) HbA_1c_ 51- and 275-SNP instrument and partitioned glycaemic/erythrocytic instruments

As per Table 1 below, using 51 and 275 HbA_1c_ SNPs in UKB, the ‘Individual-level data regression’ and Cragg-Donald formulae gave similar F-statistics (using the same R^2^ values of 2.8% and 3%). The two methods yielded somewhat different F-statistics for the 16-SNP glycaemic instrument, but both were substantially larger than 10, indicating no cause for concern (Table 1). For the 19-SNP erythrocytic instrument the F-statistics obtained using both methods were comparable (Table 1).

### Type-2 diabetes 157-SNP instrument in UKB

Table 1 presents F-statistics and R^2^ metrics using both methods. Results were comparable irrespective of which formula was used (with the same R^2^ of 1.5%).

### Which approach should I use in my study?

The ‘Individual-level data regression’ approach naturally requires individual-level data for the exposure of interest, which are not always available to researchers. The Cragg-Donald formula, however, relies on having information about the R^2^ which could come from the published GWAS for the exposure, yet this is not always included in GWAS papers. The ‘t-statistic’ approach can be used to calculate the F-statistic when the R^2^ is not known if betas or log(betas) and standard errors are provided in the summary-level GWAS exposure dataset. Thus, if individual-level data are available then the ‘Individual-level data regression’ may be recommended, but if this is not the case then the Cragg-Donald formula can be used.

## Discussion

### Consideration of total and average instrument strength for HbA_1C_ and type-2 diabetes

Across our empirical examples in the UK Biobank, the HbA1c instrument outperformed that for type 2 diabetes, in terms of total strength (R^2^) and average strength (F-statistic) even though it contained markedly fewer SNPs. Specifically, the 16-SNP glycaemic instrument had the highest average strength and explained 1% of the variance in HbA_1c_, which is lower than the 2.8% variance explained for the 51-SNP instrument, but certainly still appropriate for use in MR. The type-2 diabetes 157-SNP instrument had a much smaller F-statistic (F<30) in UKB overall and explained around 1.5% of the variance in diabetes in UKB. On the other hand, the HbA_1c_ erythrocytic instrument also demonstrated that it is more than adequate for use in MR studies, with a similar R^2^ to the glycaemic variants and an F value of just under 200. Therefore, whether it is partitioned into glycaemic and erythrocytic or not the HbA1c genetic instrument with 51 SNPs is overall, a strong instrument for use in MR studies, as indicated by both R^2^ and F-statistic metrics, even in comparison to the newer 275-SNP HbA_1c_ instrument. However, the type-2 diabetes instrument appears to be somewhat weaker both in terms of total and average strength, when compared to the HbA1c genetic instrument(s).

### Potential recommendations for MR studies instrumenting diabetes and/or HbA_1c_

First, as demonstrated in our empirical examples and argued above, ‘bigger is not always better’ when it comes to selection of instruments for glycaemic MR studies. Above we show that in some cases glycaemic instruments with fewer SNPs may be stronger and thus, more robust for use in MR when it comes to trying to minimise the important issue of ‘weak instrument bias’. This is the case for both HbA_1c_ and diabetes, with the HbA_1c_ instrument being superior. We therefore recommend that researchers do not assume that the latest and largest GWAS will always yield the best genetic instrument for these exposures and that careful consideration should be given to which GWAS is selected for the exposure. Genetic variants identified in older GWA studies may of course also be pleiotropic. Thus, researchers might choose to empirically test this in their MR study by for example, performing a Phenome-Wide Association Study (PheWAS). However, it is important to note that instrument selection will likely have to balance choosing an instrument with a larger number of genetic variants (greater R^2^=total strength), but potentially with smaller average strength (lower F-statistic). When prioritising the former, it is more likely that the instrument will include pleiotropic variants, which violates a core MR assumption. If the latter is prioritised it is possible that the total instrument strength may be weakened, as fewer variants often yield a larger F-statistic, but with lower variance explained in the exposure (R^2^). However, it is also important to note that more variants provide opportunities to run more robust methods, including common sensitivity analyses such as the MR-Egger test. For the HbA_1c_ instrument exemplified above in the UKB cohort, however, when we partitioned by glycaemic vs. erythrocytic variants the R^2^ remained at 1% for a small number of SNPs. Therefore, this example is a demonstration of an integrated approach that considers the total and average strength of the instrument, alongside biological function of the variants. In addition, another way to avoid pleiotropy is to use an approach such as that of Luo and colleagues (75), who adjusted for erythrocytic properties to control for unknown sources of pleiotropy.

Second, to reiterate the recommendation made by Boef and colleagues in 2015, and the more recent STROBE-MR guidelines (66), authors of MR studies should calculate and report the F-statistic for the association between their genetic instrument and the exposure of interest in their study. As demonstrated earlier, this can be calculated using one of three approaches, depending on whether researchers have access to individual-level data or not. If individual-level data are available for the exposure of interest, then researchers should likely prioritise calculating the F-statistic using the ‘Individual-level data regression’ approach. If individual-level data are not accessible, but the exposure GWAS paper provides the R^2^ for the (exact) instrument that is being used, then we recommend using the Cragg-Donald F-statistic method. An additional method exists, namely the ‘t-statistic’ method, which we did not implement here. This is because the ‘t-statistic’ method (F= β^2^/SE^2^) can be used when the R^2^ is not known (i.e., not provided in the paper for the GWAS for the exposure). In this equation, *β* represents the coefficient for each SNP’s association with the exposure and SE its standard error. Using the ‘t-statistic’ method the obtained F-statistic will be more of an approximation because it uses the discovery GWAS (usually for the exposure) sample size, rather than that of the outcome dataset.

Third, and related to our earlier point, there are some complex issues surrounding genetic instrumentation of binary disease exposures such as diabetes (76,77). When instrumenting these types of disease exposures, it is important to note that we are modelling an underlying continuous measure where liability thresholds are used to separate individuals into different categories (76,78) and we should thus, interpret MR using binary exposures in terms of genetic liability (78). If MR instrumental variable assumptions are met for the underlying continuous exposure which is used to categorise individuals, then we assume that we can infer causality using the binary exposure (76). However, there may be circumstances in which researchers feel the need to genetically instrument diabetes itself as it may prove to be clinically informative. We would still recommend that researchers interested in how hyperglycaemia might causally impact a range of important health outcomes, take advantage of what is evidently a strong HbA_1c_ instrument. This instrument is currently underused, as we found only 22 studies that used it as an exposure in MR studies and thus, we recommend that researchers exploit this instrument to a much greater extent. Also, the MAGIC Consortium GWA studies do not include UKB making this instrument very attractive for use in two-sample MR studies of HbA_1c_ and important health outcomes. In terms of instrument metrics, our applied example in UKB data clearly showed that the HbA_1c_ instrument completely outperformed the diabetes instrument. The HbA_1c_ instrument can also be split by biological function, into erythrocytic and glycaemic SNPs, as shown above in our examples. Genetic instrumentation of a continuous exposure such as HbA_1c_ also enables the application of non-linear MR methods (79), which are also somewhat underused in MR. Using non-linear MR methods can help define levels of risk and may also aid in understanding that it is both low and high levels of HbA1c that are associated with risk. While understanding the causal impact of disease status (e.g., diabetes) on a range of outcomes is both interesting and important, it is well established that continuous measures are superior and should be used where possible.

Fourth, we recommend that where plausible, researchers may adopt an instrument selection approach such as that of Burgess et al(57) which we described earlier (Example A) with the aim of illustrating a novel line of thinking to integrate both diabetes and HbA_1c_ into an MR study. This study used a method which exploited properties of each of these exposures and this yielded an instrument with good average strength (F=144) and total strength (2.8% variance explained). An alternative form of biological integration is illustrated in the work of Yeung and colleagues (80), and Yuan et al (81) who integrated expression of relevant genes and HbA_1c_ in their instrument selection process.

Fifth, another example of an integrated approach to instrument selection is provided in Example B above, in which we sought to bypass the issue of sample overlap in our previous MR study. To try to mitigate this we took as many of the newer diabetes variants as possible (from a more recent GWAS, but that contained overlap with our data under study) and used the effect estimates from the earlier GWAS. The most popular approach to instrument selection is to naturally take the most recent, largest GWAS (which often includes UKB), due to assumptions that the benefits (e.g., large number of genetic variants) outweigh the risks (e.g., sample overlap). However, we show that a diabetes instrument with 77 SNPs had a larger F-statistic (average strength) indicating that if anything, this instrument carried a lower risk of weak instrument bias compared to our original 157-SNP instrument.

While our paper focuses on genetic instrument selection for MR studies of HbA_1c_ and/or liability to diabetes, we acknowledge that as a method, MR has limitations and is not a panacea for causality. As such, triangulation of findings is crucial whereby different study designs are employed to be enable robust causal statements. Key limitations of MR include confounding by ancestry, confounding by linkage disequilibrium (LD), confounding by horizontal pleiotropy and canalisation (82). Confounding by ancestry, or population stratification, refers to the fact that allele frequencies of common genetic variants, as well as disease frequencies, may differ by population. However, it is now common to adjust for genetic principal components in MR studies to correct for residual confounding by population structure. Confounding by LD refers to when the selected genetic variant(s) is/are in LD (i.e., correlated with) another genetic variant associated with the outcome under study, which may produce a confounded causal estimate. Confounding by horizontal pleiotropy is when a single genetic variant influences the outcome under study directly, rather than via the exposure being instrumented. However, numerous methods have been developed to detect and correct for horizontal pleiotropy (83). Canalisation is when an individual develops a compensatory mechanism for disruptive genetic or environmental influences, as a response to higher or lower levels of a risk factor (e.g., higher, or lower body mass index).

## Conclusions

In summary, we recommend that MR studies of glycaemia take a more integrated approach when it comes to selection of genetic instruments. Therefore, careful consideration should be given to the following: i) whether novel approaches such as those described here from the literature might be used; ii) which GWAS is used to select the instrument for the exposure; iii) whether a continuous, as opposed to a binary exposure can be instrumented; iv) inclusion of both variance explained (R^2^=total strength of the instrument) and the F-statistic (average strength).

## Supplementary Material

Supplementary material

## Figures and Tables

**Table 1:** Instrument strength metrics in UKB (N=349,326)

Trait	Variance explained (R^2^)	F-statistic	Method
**Diabetes (157 SNPs)**	0.015 (1.5%) 0.015 (1.5%)	27.43 27.9	ILDR CD
**HbA_1c_ main instrument (51-SNPs)**.	0.028 (2.8%) 0.028 (2.8%)	164.6 164.8	ILDR CD
**HbA1c main instrument (275 SNPs)**	0.030 (3%) 0.030 (3%)	33.24. 38.08	ILDR CD
**HbA_1c_ 16-SNP glycaemic instrument**	0.011 (1.1%) 0.011 (1.1%)	201.1 182.3	ILDR CD
**HbA_1c_ 19-SNP erythrocytic instrument**	0.012 (1.2%) 0.012 (1.2%)	187.5 184.3	ILDR CD

Note. ILDR=‘individual-level data regression’, CD=Cragg-Donald.

## Data Availability

The UK Biobank data are available via: www.ukbiobank.ac.uk/using-the-resource/. This study was conducted using the UK Biobank Resource Application ID 7661.

## Research In Context

### What is already known about this subject?

- Mendelian randomisation studies of glycaemia have become particularly popular in recent years.
- Genetic instrument selection strategies are often suboptimal and poorly reported in studies seeking to understand causality of HbA_1c_/diabetes and important outcomes.

### What is the key question?

- What strategies are currently employed in glycaemic MR studies when it comes to genetic instrument selection?

### What are the new findings?

- Far more MR studies instrument diabetes, as opposed to HbA1c, as revealed by our literature search.
- However, our empirical examples in the UK Biobank showed that an HbA1c genetic instrument is likely superior to a diabetes one in terms of total and average strength, even when partitioned by biological function.
- Importantly, though, the diabetes genetic instrument performed well and we are aware that in certain scenarios researchers prefer to instrument a binary exposure, such as diabetes.

### How might this impact on clinical practice in the foreseeable future?

- MR studies to date may have had discrepant findings, due to a suboptimal instrument selection approach and our careful set of guidelines provided here will help prevent this in future studies.

## References

[1] Ahmad OS, Morris JA, Mujammami M, Forgetta V, Leong A, Li R (2015). A Mendelian randomization study of the effect of type-2 diabetes on coronary heart disease. Nat Commun.

[2] Walter S, Marden JR, Kubzansky LD, Mayeda ER, Crane PK, Chang SC (2016). Diabetic Phenotypes and Late-Life Dementia Risk. Alzheimer Dis Assoc Disord.

[3] Xu M, Huang Y, Xie L, Peng K, Ding L, Lin L (2016). Diabetes and risk of arterial stiffness: A mendelian randomization analysis. Diabetes.

[4] Xu M, Bi Y, Huang Y, Xie L, Hao M, Zhao Z (2016). Type 2 Diabetes, Diabetes Genetic Score and Risk of Decreased Renal Function and Albuminuria: A Mendelian Randomization Study. EBioMedicine.

[5] Ahmad OS, Leong A, Miller JA, Morris JA, Forgetta V, Mujammami M (2017). A Mendelian Randomization Study of the Effect of Type-2 Diabetes and Glycemic Traits on Bone Mineral Density. Journal of Bone and Mineral Research.

[6] Carreras-Torres R, Johansson M, Gaborieau V, Haycock PC, Wade KH, Relton CL (2017). The Role of Obesity, Type 2 Diabetes, and Metabolic Factors in Pancreatic Cancer: A Mendelian Randomization Study. J Natl Cancer Inst.

[7] Gan W, Clarke RJ, Mahajan A, Kulohoma B, Kitajima H, Robertson NR (2017). Bone mineral density and risk of type 2 diabetes and coronary heart disease: A Mendelian randomization study. Wellcome Open Res.

[8] Hagenaars SP, Gale CR, Deary IJ, Harris SE (2017). Cognitive ability and physical health: A Mendelian randomization study. Sci Rep.

[9] Larsson SC, Scott RA, Traylor M, Langenberg CC, Hindy G, Melander O (2017). Type 2 diabetes, glucose, insulin, BMI, and ischemic stroke subtypes: Mendelian randomization study. Neurology.

[10] Van’t Hof FNG, Vaucher J, Holmes Mv, de Wilde A, Baas AF, Blankensteijn JD (2017). Genetic variants associated with type 2 diabetes and adiposity and risk of intracranial and abdominal aortic aneurysms. European Journal of Human Genetics.

[11] Disney-Hogg L, Sud A, Law PJ, Cornish AJ, Kinnersley B, Ostrom QT (2018). Influence of obesity-related risk factors in the aetiology of glioma. Br J Cancer.

[12] Xuan L, Zhao Z, Jia X, Hou Y, Wang T, Li M (2018). Type 2 diabetes is causally associated with depression: a Mendelian randomization analysis. Front Med.

[13] Beijer K, Nowak C, Sundström J, Ärnlöv J, Fall T, Lind L (2019). In search of causal pathways in diabetes: a study using proteomics and genotyping data from a cross-sectional study. Diabetologia.

[14] Bovijn J, Jackson L, Censin J, Chen CY, Laisk T, Laber S (2019). GWAS Identifies Risk Locus for Erectile Dysfunction and Implicates Hypothalamic Neurobiology and Diabetes in Etiology. Am J Hum Genet.

[15] Funck-Brentano T, Nethander M, Movérare-Skrtic S, Richette P, Ohlsson C (2019). Causal Factors for Knee, Hip, and Hand Osteoarthritis: A Mendelian Randomization Study in the UK Biobank. Arthritis Rheumatol.

[16] Marouli E, del Greco MF, Astley CM, Yang J, Ahmad S, Berndt SI (2019). Mendelian randomisation analyses find pulmonary factors mediate the effect of height on coronary artery disease. Commun Biol.

[17] Sun D, Zhou T, Heianza Y, Li X, Fan M, Fonseca VA (2019). Type 2 Diabetes and Hypertension: A Study on Bidirectional Causality. Circ Res.

[18] Yarmolinsky J, Relton CL, Lophatananon A, Muir K, Menon U, Gentry-Maharaj A (2019). Appraising the role of previously reported risk factors in epithelial ovarian cancer risk: A Mendelian randomization analysis. PLoS Med.

[19] Au Yeung SL, Schooling CM (2019). Impact of glycemic traits, type 2 diabetes and metformin use on breast and prostate cancer risk: A Mendelian randomization study. BMJ Open Diabetes Res Care.

[20] Yeung CHC, Au Yeung SL, Fong SSM, Schooling CM (2019). Lean mass, grip strength and risk of type 2 diabetes: a bi-directional Mendelian randomisation study. Diabetologia.

[21] Zeng P, Wang T, Zheng J, Zhou X (2019). Causal association of type 2 diabetes with amyotrophic lateral sclerosis: new evidence from Mendelian randomization using gWaS summary statistics. BMC Med.

[22] Bell JA, Bull CJ, Gunter MJ, Carslake D, Mahajan A, Smith GD (2020). Early metabolic features of genetic liability to type 2 diabetes: Cohort study with repeated metabolomics across early life. Diabetes Care.

[23] Gill D, Arvanitis M, Carter P, Hernández Cordero AI, Jo B, Karhunen V (2020). ACE inhibition and cardiometabolic risk factors, lung ACE2 and TMPRSS2 gene expression, and plasma ACE2 levels: A Mendelian randomization study: ACE inhibition and ACE2 expression. R Soc Open Sci.

[24] Elhadad MA, Jonasson C, Huth C, Wilson R, Gieger C, Matias P (2020). Deciphering the plasma proteome of type 2 diabetes. Diabetes.

[25] Gudmundsdottir V, Zaghlool SB, Emilsson V, Aspelund T, Ilkov M, Gudmundsson EF (2020). Circulating Protein Signatures and Causal Candidates for Type 2 Diabetes. Diabetes.

[26] Harrison S, Davies AR, Dickson M, Tyrrell J, Green MJ, Katikireddi SV (2020). The causal effects of health conditions and risk factors on social and socioeconomic outcomes: Mendelian randomization in UK Biobank. Int J Epidemiol.

[27] Inamo J, Kochi Y, Takeuchi T (2021). Is type 2 diabetes mellitus an inverse risk factor for the development of rheumatoid arthritis?. J Hum Genet.

[28] Kwok MK, Kawachi I, Rehkopf D, Schooling CM (2020). The role of cortisol in ischemic heart disease, ischemic stroke, type 2 diabetes, and cardiovascular disease risk factors: a bi-directional Mendelian randomization study. BMC Med.

[29] Lu Y, Gentiluomo M, Lorenzo-Bermejo J, Morelli L, Obazee O, Campa D (2020). Mendelian randomisation study of the effects of known and putative risk factors on pancreatic cancer. J Med Genet.

[30] Pan Y, Chen W, Yan H, Wang M, Xiang X (2020). Glycemic traits and Alzheimer’s disease: a Mendelian randomization study. Aging.

[31] Parisinos CA, Wilman HR, Thomas EL, Kelly M, Nicholls RC, McGonigle J (2020). Genome-wide and Mendelian randomisation studies of liver MRI yield insights into the pathogenesis of steatohepatitis. J Hepatol.

[32] Rao S, Lau A, So HC (2020). Exploring Diseases/Traits and Blood Proteins Causally Related to Expression of ACE2, the Putative Receptor of SARS-CoV-2: A Mendelian Randomization Analysis Highlights Tentative Relevance of Diabetes-Related Traits. Diabetes Care.

[33] Smit RAJ, Trompet S, Leong A, Goodarzi MO, Postmus I, Warren H (2020). Statin-induced LDL cholesterol response and type 2 diabetes: a bidirectional two-sample Mendelian randomization study. Pharmacogenomics J.

[34] Tang B, Yuan S, Xiong Y, He Q, Larsson SC (2020). Major depressive disorder and cardiometabolic diseases: a bidirectional Mendelian randomisation study. Diabetologia.

[35] Thomassen JQ, Tolstrup JS, Benn M, Frikke-Schmidt R (2020). Type-2 diabetes and risk of dementia: observational and Mendelian randomisation studies in 1 million individuals. Epidemiol Psychiatr Sci.

[36] van Oort S, Beulens JWJ, van Ballegooijen AJ, Burgess S, Larsson SC (2021). Cardiovascular risk factors and lifestyle behaviours in relation to longevity: a Mendelian randomization study. J Intern Med.

[37] Wang N, Wang C, Chen X, Wan H, Chen Y, Chen C (2020). Vitamin D prediabetes and type 2 diabetes: bidirectional Mendelian randomization analysis. Eur J Nutr.

[38] Yuan S, Larsson SC (2020). An atlas on risk factors for type 2 diabetes: a wideangled Mendelian randomisation study. Diabetologia.

[39] Andrews SJ, Fulton-Howard B, O’Reilly P, Marcora E, Goate AM, Farrer LA (2021). Causal Associations Between Modifiable Risk Factors and the Alzheimer’s Phenome. Ann Neurol.

[40] Cui Z, Feng H, He B, Xing Y, Liu Z, Tian Y (2021). Type 2 Diabetes and Glycemic Traits Are Not Causal Factors of Osteoarthritis: A Two-Sample Mendelian Randomization Analysis. Front Genet.

[41] Jones G, Trajanoska K, Santanasto AJ, Stringa N, Kuo CL, Atkins JL (2021). Genome-wide meta-analysis of muscle weakness identifies 15 susceptibility loci in older men and women. Nat Commun.

[42] Peters TM, Holmes Mv, Richards JB, Palmer T, Forgetta V, Lindgren CM (2021). Sex Differences in the Risk of Coronary Heart Disease Associated With Type 2 Diabetes: A Mendelian Randomization Analysis. Diabetes Care.

[43] Molina-Montes E, Coscia C, Gómez-Rubio P, Fernández A, Boenink R, Rava M (2021). Deciphering the complex interplay between pancreatic cancer, diabetes mellitus subtypes and obesity/BMI through causal inference and mediation analyses. Gut.

[44] Yuan S, Xiong Y, Larsson SC (2021). An atlas on risk factors for multiple sclerosis: a Mendelian randomization study. J Neurol.

[45] Yuan S, Giovannucci EL, Larsson SC (2021). Gallstone disease, diabetes, calcium, triglycerides, smoking and alcohol consumption and pancreatitis risk: Mendelian randomization study. NPJ Genom Med.

[46] Au Yeung SL, Luo S, Schooling CM (2018). The Impact of Glycated Hemoglobin (HbA(1c)) on Cardiovascular Disease Risk: A Mendelian Randomization Study Using UK Biobank. Diabetes Care.

[47] Hsiung CN, Chang YC, Lin CW, Chang CW, Chou WC, Chu HW (2020). The Causal Relationship of Circulating Triglyceride and Glycated Hemoglobin: A Mendelian Randomization Study. Journal of Clinical Endocrinology and Metabolism.

[48] Jia X, Hou Y, Xu M, Zhao Z, Xuan L, Wang T (2019). Mendelian Randomization Analysis Support Causal Associations of HbA1c with Circulating Triglyceride, Total and Low-density Lipoprotein Cholesterol in a Chinese Population. Sci Rep.

[49] Leong A, Chen J, Wheeler E, Hivert MF, Liu CT, Merino J (2019). Mendelian Randomization Analysis of Hemoglobin A(1c) as a Risk Factor for Coronary Artery Disease. Diabetes Care.

[50] Liu HM, Hu Q, Zhang Q, Su GY, Xiao HM, Li BY (2019). Causal effects of genetically predicted cardiovascular risk factors on chronic kidney disease: A two-sample mendelian randomization study. Front Genet.

[51] Aung N, Khanji MY, Munroe PB, Petersen SE (2020). Causal Inference for Genetic Obesity, Cardiometabolic Profile and COVID-19 Susceptibility: A Mendelian Randomization Study. Front Genet.

[52] Dikilitas O, Satterfield BA, Kullo IJ (2020). Risk factors for polyvascular involvement in patients with peripheral artery disease: A mendelian randomization study. J Am Heart Assoc.

[53] Hu X, Zhuang XD, Mei Wy, Liu G, Du ZM, Liao XX (2020). Exploring the causal pathway from body mass index to coronary heart disease: a network Mendelian randomization study. Ther Adv Chronic Dis.

[54] Jin H, Lee S, Won S (2020). Causal Evaluation of Laboratory Markers in Type 2 Diabetes on Cancer and Vascular Diseases Using Various Mendelian Randomization Tools. Front Genet.

[55] Au Yeung SL, Luo S, Schooling CM (2020). The impact of glycated hemoglobin on risk of hypertension: a Mendelian randomization study using UK Biobank. J Hypertens.

[56] Zhao Jv, Schooling CM (2020). Sex-specific associations of insulin resistance with chronic kidney disease and kidney function: a bi-directional Mendelian randomisation study. Diabetologia.

[57] Burgess S, Malik R, Liu B, Mason AM, Georgakis Mk, Dichgans M (2021). Dose-response relationship between genetically proxied average blood glucose levels and incident coronary heart disease in individuals without diabetes mellitus. Diabetologia.

[58] Juvinao-Quintero DL, Starling AP, Cardenas A, Powe CE, Perron P, Bouchard L (2021). Epigenome-wide association study of maternal hemoglobin A1c in pregnancy and cord blood DNA methylation. Epigenomics.

[59] Saunders CN, Cornish AJ, Kinnersley B, Law PJ, Houlston RS, Claus EB (2021). Searching for causal relationships of glioma: a phenome-wide Mendelian randomisation study. Br J Cancer.

[60] Davey Smith G, Ebrahim S (2003). ‘Mendelian randomization’: can genetic epidemiology contribute to understanding environmental determinants of disease?. Int J Epidemiol.

[61] Wheeler E, Leong A, Liu CT, Hivert MF, Strawbridge RJ, Podmore C (2017). Impact of common genetic determinants of Hemoglobin A1c on type 2 diabetes risk and diagnosis in ancestrally diverse populations: A transethnic genome-wide meta-analysis. PLoS Med.

[62] Mahajan A, Taliun D, Thurner M, Robertson NR, Torres JM, Rayner NW (2018). Fine-mapping type 2 diabetes loci to single-variant resolution using high-density imputation and islet-specific epigenome maps. Nat Genet.

[63] Xue A, Wu Y, Zhu Z, Zhang F, Kemper KE, Zheng Z (2018). Genome-wide association analyses identify 143 risk variants and putative regulatory mechanisms for type 2 diabetes. Nat Commun.

[64] Vujkovic M, Keaton JM, Lynch JA, Miller DR, Zhou J, Tcheandjieu C (2020). Discovery of 318 new risk loci for type 2 diabetes and related vascular outcomes among 1.4 million participants in a multi-ancestry meta-analysis. Nat Genet.

[65] Chen J, Spracklen CN, Marenne G, Varshney A, Corbin LJ, Luan J (2021). The trans-ancestral genomic architecture of glycemic traits. Nat Genet.

[66] Skrivankova VW, Richmond RC, Woolf BAR, Davies NM, Swanson SA, Vanderweele TJ (2021). Strengthening the reporting of observational studies in epidemiology using mendelian randomisation (STROBE-MR): Explanation and elaboration. The BMJ.

[67] Lawlor DA (2016). Commentary: Two-sample Mendelian randomization: opportunities and challenges. Int J Epidemiol.

[68] Burgess S, Thompson SG (2011). Avoiding bias from weak instruments in mendelian randomization studies. Int J Epidemiol.

[69] Garfield V, Farmaki AE, Fatemifar G, Eastwood Sv, Mathur R, Rentsch CT (2021). The Relationship Between Glycaemia, Cognitive Function, Structural Brain Outcomes and Dementia: A Mendelian Randomisation Study in the UK Biobank. Diabetes.

[70] Consortium DiaGRAM analysis (DIAGRAM), Consortium AGENT 2 D (AGEN T, Consortium SAT 2 D (SAT2D), Consortium MAT 2 D (MAT2D), Consortium T 2 DGE by N generation sequencing in muylti ES (T2D G, Mahajan A (2014). Genome-wide trans-ancestry meta-analysis provides insight into the genetic architecture of type 2 diabetes susceptibility. Nat Genet.

[71] Larsson SC, Bäck M, Rees JMB, Mason AM, Burgess S (2020). Body mass index and body composition in relation to 14 cardiovascular conditions in UK Biobank: a Mendelian randomization study. Eur Heart J.

[72] Zhu P, Herrington WG, Haynes R, Emberson J, Landray MJ, Sudlow CLM (2021). Conventional and Genetic Evidence on the Association between Adiposity and CKD. J Am Soc Nephrol.

[73] Sudlow C, Gallacher J, Allen N, Beral V, Burton P, Danesh J (2015). UK Biobank: An Open Access Resource for Identifying the Causes of a Wide Range of Complex Diseases of Middle and Old Age. PLoS Med.

[74] Mahajan A, Taliun D, Thurner M, Robertson NR, Torres JM, Rayner NW (2018). Fine-mapping type 2 diabetes loci to single-variant resolution using high-density imputation and islet-specific epigenome maps. Nat Genet.

[75] Luo S, Au Yeung SL, Schooling CM (2021). Assessing the linear and non-linear association of HbA1c with cardiovascular disease: a Mendelian randomisation study. Diabetologia.

[76] Burgess S, Labrecque JA (2018). Mendelian randomization with a binary exposure variable: interpretation and presentation of causal estimates. Eur J Epidemiol.

[77] Howe LJ, Tudball M, Smith GD, Davies NM (2020). Interpreting mendelian randomization estimates of the effects of categorical exposures such as disease status and educational attainment. medRxiv.

[78] Howe LJ, Tudball M, Davey Smith G, Davies NM (2021). Interpreting Mendelian-randomization estimates of the effects of categorical exposures such as disease status and educational attainment. Int J Epidemiol.

[79] Staley JR, Burgess S (2017). Semiparametric methods for estimation of a nonlinear exposure-outcome relationship using instrumental variables with application to Mendelian randomization. Genet Epidemiol.

[80] Yuan S, Mason AM, Burgess S, Larsson SC (2022). Differentiating Associations of Glycemic Traits with Atherosclerotic and Thrombotic Outcomes: Mendelian Randomization Investigation. Diabetes.

[81] Au Yeung SL, Zhao Jv, Schooling CM (2021). Evaluation of glycemic traits in susceptibility to COVID-19 risk: a Mendelian randomization study. BMC Med.

[82] Smith GD, Ebrahim S (2004). Mendelian randomization: prospects, potentials, and limitations. Int J Epidemiol.

[83] Davies NM, Holmes Mv, Davey Smith G (2018). Reading Mendelian randomisation studies: A guide, glossary, and checklist for clinicians. BMJ.

