# Supplementary material for: A Guide for Selection of Genetic Instruments in Mendelian randomisation (MR) studies of Type-2 diabetes and HbA1c: towards an integrated approach
Source: Diabetes. Author manuscript; Available in PMC 2023 May 30. (PMC7614590; doi:10.2337/db22-0110)
Supplement: Supplementary material [file EMS175064-supplement-Supplementary_material.pdf]

Supplemental material S1

Search strategy

Diabetes searches in PubMed

We conducted searches in PubMed using the following terms, until March 2021: "mendelian randomization"[Title/Abstract] AND "diabetes"[Title/Abstract], and "diabetes"[Title/Abstract] AND "mendelian randomisation"[Title/Abstract].

HbA_{1c} searches in PubMed

We conducted the following searches for HbA_{1c} MR studies: (((HbA1c) AND (Mendelian randomization)) OR (HbA1c)) AND (Mendelian randomisation); (("HbA1c"[Title/Abstract] AND "mendelian randomisation"[Title/Abstract]) OR "HbA1c"[Title/Abstract]) AND "mendelian randomization"[Title/Abstract]; and (("glycated haemoglobin"[Title/Abstract] AND "mendelian randomisation"[Title/Abstract]) OR "glycated haemoglobin"[Title/Abstract]) AND "mendelian randomization"[Title/Abstract].

Additional searches in Web of Science

After screening it was found that all studies had either been identified through the PubMed search or were not appropriate for inclusion due to having not instrumented diabetes. The search for diabetes was: TITLE: (diabetes* AND Mendelian randomisation). Refined by: DOCUMENT TYPES: (ARTICLE). For HbA_{1c} it was: TITLE: ($HbA1_c$* AND Mendelian randomisation). Refined by: DOCUMENT TYPES: (ARTICLE). After filtering out conference abstracts there was only one article that had already been identified in the PubMed search.

Figure 1. Flowchart for diabetes literature search (using PRISMA guidelines)

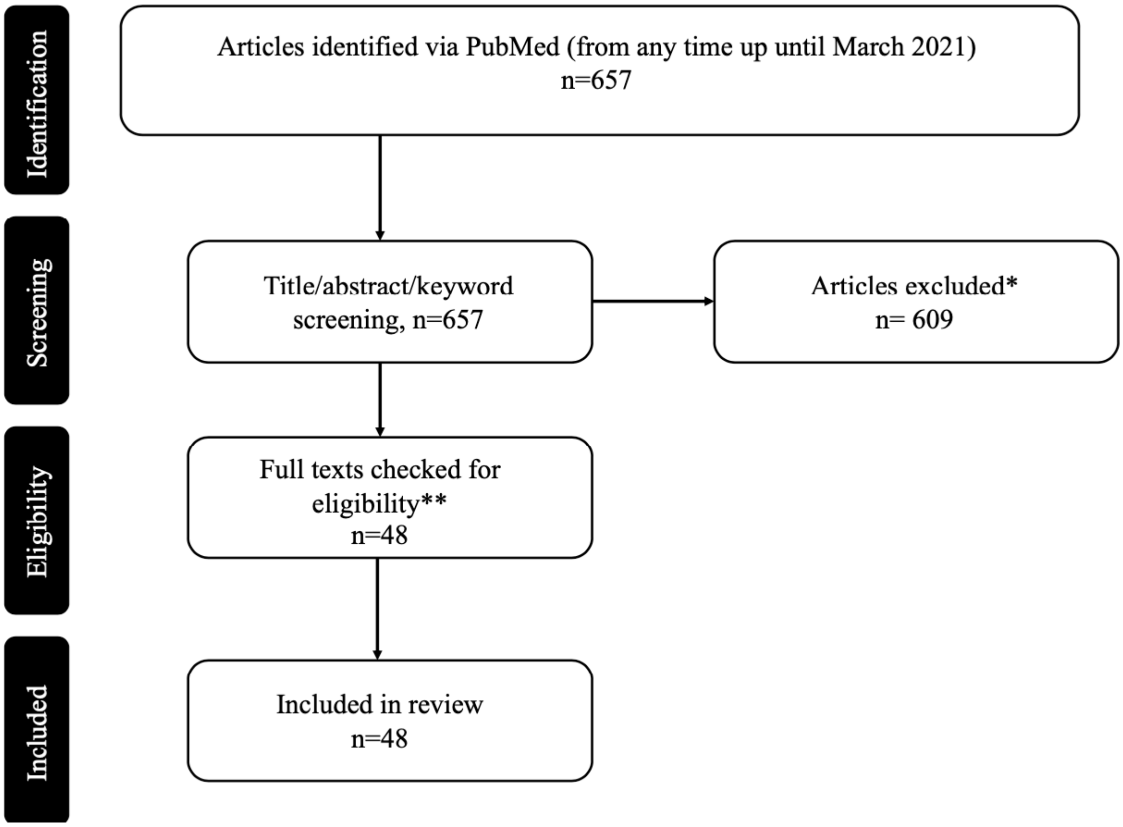

Note. *Excluded because it was evident from the title/abstract/keywords that they did not instrument diabetes as an exposure or because it was a duplicate; **confirmation that diabetes was instrumented using MR and that details were provided on the number of SNPs used and details of which GWAS they were discovered in.

Figure 2. Flowchart for HbA_{1c} literature search (using PRISMA guidelines)

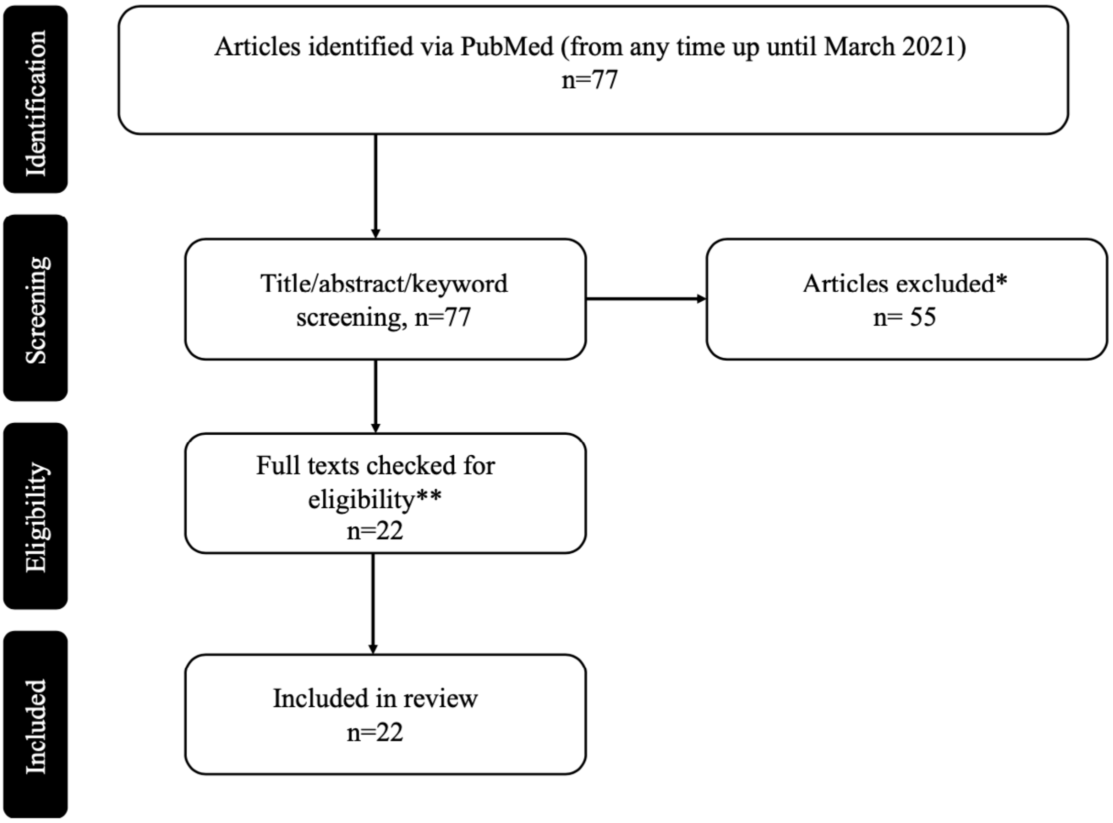

Note. *Excluded because it was evident from the title/abstract/keywords that they did not instrument HbA_{1c} as an exposure or because it was a duplicate.; **confirmation that HbA_{1c} was instrumented using MR and that details were provided on the number of SNPs used and details of which GWAS they were discovered in.

Table 1. MR studies until March 2021 that used diabetes as an (genetically instrumented) exposure

First author (year)	PubMed ID	Outcome
Ahmad (2015)	26017687	Coronary Heart Disease (CHD)
Walter (2016)	26650880	Alzheimer's dementia (AD)
Xu (2016)	26953161	Arterial stiffness
Xu (2016)	27211558	Renal function & albuminuria
Ahmad (2017)	27982478	Bone mineral density
Carreras-Torres (2017)	28954281	Pancreatic cancer
Gan (2017)	28989980	Bone mineral density
Hagenaars (2017)	28572633	Cognitive ability
Larsson (2017)	28667182	Stroke
Van 't Hof (2017)	28378816	Intracranial and abdominal aortic aneurysms
Disney-Hogg (2018)	29531326	Glioma
Wang (2018)	29506267	Lipid profiles
Xuan (2018)	30446878	Depression
Beijer (2019)	31446444	Plasma proteins
Bovijn (2019)	30583798	Erectile dysfunction
Funck-Brentano (2019)	31099188	Osteoarthritis
Marouli (2019)	30937401	CAD
Sun (2019)	30646822	Hypertension
Yarmolinksy (2019)	31390370	Epithelial ovarian cancer
Yeung (2019)	31908803	Cancers
Yeung (2019)	30798333	Lean mass/grip strength
Zeng (2019)	31796040	Amyotrophic lateral sclerosis (ALS)
Bell (2020)	32345654	NMR metabolites
Gill (2020)	33391794	Lung ACE2 and TMPRSS2 expression and ACE2 plasma levels
Goto (2020)	30927373	Cancer risk
Elhadad (2020)	32928870	Plasma proteins
Gudmundsdottir (2020)	32385057	Serum proteins
Harrison (2020)	32808034	Social and SES outcomes
Inamo (2020)	32901113	Rheumatoid arthritis
Kwok (2020)	33243239	Cortisol
Lu (2020)	32066631	Pancreatic cancer
Pan (2020)	33202379	AD
Parisinos (2020)	32247823	Liver cT1 measures
Rao (2020)	32430459	ACE2 expression
Smit (2020)	31801993	Statin-induced LDL cholesterol response
Tang (2020)	32270255	Major depressive disorder (MDD)
Thomassen (2020)	32326995	Dementia
Van Oort (2020)	33131310	Hypertension
Wang (2020)	31076857	Vitamin D
Yuan (2020)	32349989	Cancers (x22)
Andrews (2021)	32996171	Alzheimer's phenome
Cui (2021)	33519901	Osteoarthritis
Jones (2021)	33510174	Muscle weakness
Peters (2021)	33277303	CHD
Molina-Montes (2021)	32409590	Pancreatic cancer
Van Oort (2021)	33107078	Longevity
Yuan (2021)	33418132	Gallstone disease
Yuan (2021)	32728946	Multiple sclerosis

Table 2. MR studies until March 2021 that used HbA_{1c} as an (genetically instrumented) exposure

First author (year)	PubMed ID	Outcome
Yeung (2018)	29950300	CVD
Hsiung (2020)	31784746	Circulating triglycerides
Jia (2019)	30940890	Triglycerides, Total and LDL cholesterol
Leong (2019)	30659074	CAD
Liu (2019)	31130989	CKD
Aung (2020)	33262790	COVID-19 susceptibility
Dikilitas (2020)	33287626	Peripheral artery disease
Hu (2020)	32523662	CHD
Jin (2020)	33408737	Vascular outcomes
Yeung (2020)	31386636	Hypertension
Zhao (2020)	32409868	Kidney function
Burgess (2021)	33495845	Incident CHD
Juvinao-Quintero (2021)	33406918	Cord blood DNA
Saunders (2021)	33020596	Glioma
